# Transcriptomic Identification and Expression Profile Analysis of Odorant-Degrading Enzymes from the Asian Corn Borer Moth, *Ostrinia furnacalis*

**DOI:** 10.3390/insects13111027

**Published:** 2022-11-06

**Authors:** Liya Zhang, Yidan Shen, Xingchuan Jiang, Su Liu

**Affiliations:** 1 Anhui Provincial Key Laboratory of Integrated Pest Management on Crops, School of Plant Protection, Anhui Agricultural University, Hefei 230036, China; 2 State Key Laboratory for Biology of Plant Diseases and Insect Pests, Institute of Plant Protection, Chinese Academy of Agricultural Sciences, Beijing 100193, China

**Keywords:** *Ostrinia furnacalis*, antennal transcriptome, odorant degradation, expression profiles

## Abstract

**Simple Summary:**

*Ostrinia furnacalis*, the Asian corn borer moth, is a major insect pest of corn. The *O. furnacalis* adults use their olfactory system in complex physiological behaviors, including host search, mating, and oviposition. However, the mechanisms underlying odor signal termination in *O. furnacalis* remain largely unknown. This study aimed to unravel the odorant-degrading enzymes (ODEs) associated with odor signal inactivation in the antennae of *O. furnacalis*. By searching the antennal transcriptome, we identified a large number of genes encoding ODEs. Furthermore, the expression patterns of carboxylesterase in different adult tissues were examined to identify male- or female-biased genes. The findings of this study indicate the potential involvement of ODEs in the olfactory system of *O. furnacalis* and may help to develop novel pest management strategies.

**Abstract:**

The Asian corn borer moth *Ostrinia furnacalis* is an important lepidopteran pest of maize in Asia. Odorant-degrading enzymes (ODEs), including carboxylesterases (CCEs), glutathione *S*-transferases (GSTs), cytochrome P450s (CYPs), UDP-glycosyltransferases (UGTs), and aldehyde oxidases (AOXs), are responsible for rapid inactivation of odorant signals in the insect antennae. In this study, we performed a transcriptome assembly for the antennae of *O. furnacalis* to identify putative ODE genes. Transcriptome sequencing revealed 35,056 unigenes, and 21,012 (59.94%) of these were annotated by searching against the reference sequences in the NCBI non-redundant (NR) protein database. For functional classification, these unigenes were subjected to Gene Ontology (GO), Eukaryotic Orthologous Groups (KOG), and Kyoto Encyclopedia of Genes and Genomes (KEGG) annotations. We identified 79 genes encoding putative ODEs: 19 CCEs, 17 GSTs, 24 CYPs, 13 UGTs, and 6 AOXs. BLASTX best hit results indicated that these genes shared quite high amino acid identities with their respective orthologs from other lepidopteran species. Reverse transcription-quantitative PCR showed that *OfurCCE2*, *OfurCCE5*, and *OfurCCE18* were enriched in male antennae, while *OfurCCE7* and *OfurCCE10* were enriched in female antennae. *OfurCCE14* and *OfurCCE15* were expressed at near-equal amounts in the antennae of both sexes. Our findings establish a solid foundation for future studies aimed at understanding the olfactory functions of these genes in *O. furnacalis*.

## 1. Introduction

Olfaction is essential for an insect’s survival and reproduction. Many life activities of insects such as host search, mating, and oviposition site location rely heavily on olfactory sensation [1]. In the olfactory process, semiochemicals enter the antennal sensillum and active odorant receptors (ORs) and ionotropic receptors (IRs) [2,3]. However, after the activation the odorant molecules need to be rapidly removed to avoid receptor saturation [2]. There are various odorant-degrading enzymes (ODEs) that exist in the antennae and that are involved in the degradation of bioactive odorants into inactive compounds [4].

Several classes of ODEs have been identified in insect antennae, including carboxylesterases (CCEs), glutathione *S*-transferases (GSTs), cytochrome P450s (CYPs), UDP-glycosyltransferases (UGTs), and aldehyde oxidases (AOXs) [4]. CCEs constitute a multigene family that hydrolyze carboxylic esters [5]. The first CCE with an odorant degradation function was discovered in the giant silk moth *Antheraea polyphemus* and named ApolPDE [6]. ApolPDE is a male antennae-specific CCE that can rapidly degrade the sex pheromone component, the volatile ester–E6Z11-16:OAc [6,7]. Since then, a growing number of antennal CCEs have been identified from different insect orders, and their functions in odorant clearance have been characterized. For example, in the fruit fly *Drosophila melanogaster*, two CCEs (EST-6 and JHEdup) are enriched in the antennae and have high catalytic efficiency against the ester compounds emitted by fruit [8,9]. Furthermore, in the Japanese beetle *Popilia japonica*, a male antennae-specific CCE is able to degrade the ester pheromone (*R*)-japonilure [10]. In addition, a large number of CCEs that can degrade plant esters and/or pheromonal esters were discovered in the antennae of *Spodoptera littoralis* [11,12] and *S. exigua* [13,14,15,16,17], suggesting their involvement in olfactory signal termination.

GSTs belong to a diverse family of detoxification enzymes. These enzymes can catalyze the conjugation of glutathione to electrophilic centers of non-polar compounds [18]. A number of studies have shown that GSTs are involved in olfactory signal termination in the antennae. In the tobacco hornworm *Manduca sexta*, a GST (GST-msolf1) is localized in the pheromone-sensitive sensilla and inactivates *trans*-2-hexenal, a plant-produced green leaf aldehyde that triggers the olfactory response [19]. Moreover, in the silkworm moth *Bombyx mori*, a GST (BmGSTD4) showed restricted distribution in the sensillum lymph of male antennae, indicating that the enzyme may degrade sex pheromones [20]. A recent report showed that a GST (GmolGSTD1) is involved in the metabolism of sex pheromones and plant volatile components in the antennae of the oriental fruit moth *Grapholita molesta* [21].

Insect CYPs also belong to a diverse superfamily with detoxification functions. They play crucial roles in the detoxification of a great range of xenobiotic and endobiotic compounds, including odorants [22]. Biochemical studies provided convincing evidence that an antennae-specific CYP from the pale brown chafer *Phyllopertha diversa* can rapidly degrade the sex pheromone [23], and inhibition of the CYP activity led to pheromone anosmia in this insect species [24]. In other insect species, such as *Dendroctonus ponderosae*, *S. litura*, and *Apis mellifera*, CYPs were found to be involved in the degradation of sex pheromone constituents or phytochemical compounds in the antennae [25,26,27].

UGTs mediate the transfer of glycosyl residues from a sugar donor to a variety of harmful hydrophobic chemicals, making them more water-soluble and easier to be excreted [28]. UGTs have been demonstrated to have important roles in olfaction. Overexpression of *UGT36E1* in *D. melanogaster* antennae affected the ability to discriminate pheromones in male flies, while the ability was improved when associated with the downregulation of *UGT36E1* expression [29]. Furthermore, antennae-specific or antennae-biased expression of UGTs have been found in *D. melanogaster* [9], *Plutella xylostella* [30], and *Athetis lepigone* [31], suggesting an olfactory function of these genes.

In addition, AOXs are also associated with odorant degradation. In *A. polyphemus* and *B. mori*, antennae-specific AOXs were involved in the degradation of aldehydic sex pheromones [32]; in the navel orange worm *Amyelois transitella*, AOXs from antennae degraded both sex pheromones and plant volatile aldehydes [33]. In *Helicoverpa armigera* and *Cnaphalocrocis medinalis*, several AOXs were specifically or primarily expressed in the antennae, suggesting a putative function for degrading odorants [34,35].

The Asian corn borer moth *Ostrinia furnacalis* (Lepidoptera: Crambidae) is an important pest of maize in many Asian countries [36]. The *O. furnacalis* adults use their olfactory system to detect the chemical cues from host plants and their mates; various kinds of plant volatiles, including esters, aldehydes, alcohols, acids, terpenoids, and unsaturated hydrocarbons, can activate receptors and elicit a neuronal response that can regulate the behavior of this insect species [36]. Additionally, the female adults of *O. furnacalis* use two esters (Z12-14:OAc and E12-14:OAc) as their major sex pheromone components to attract males [37]. These odorants may enter the antennal sensilla and can be inactivated by ODEs in *O. furnacalis*. However, the molecular mechanism underlying odorant signal inactivation is still unclear in this moth species.

Transcriptome sequencing has previously been performed for the *O. furnacalis* antennae, and the repertoire of genes involved in olfaction has been identified [38]. Although most of the genes (e.g., ORs and IRs) are related to the odor recognition process, information regarding genes involved in odor degradation is still limited. Recently, Yang et al. (2015) analyzed the antennal transcriptome of *O. furnacalis* and identified a small number of genes encoding ODEs, including 15 CCEs and 8 AOXs [39]. However, this number is much less than those in other lepidopteran species such as *Cydia pomonella* [40], *Chilo suppressalis* [41], *A. lepigone* [31,42], and *S. littoralis* [43,44], suggesting that there may be additional genes that have not yet been discovered in *O. furnacalis*.

In this study, we conducted transcriptome sequencing for the *O. furnacalis* antennae and identified a total of 79 genes encoding putative ODEs, including 19 CCEs, 17 GSTs, 24 CYPs, 13 UGTs, and 6 AOXs. We also found several genes that were antennae-enriched. Our findings provide a solid foundation for studying the molecular basis of odorant signal inactivation in *O. furnacalis*.

## 2. Materials and Methods

### 2.1. Insect Rearing and Tissue Collection

The *O. furnacalis* individuals used in this study originated from a colony collected from an experimental field at Anhui Agricultural University, Hefei, China. The larvae were reared on an artificial diet, and adults were fed on a 10% (*v*/*v*) honey solution. The rearing conditions were 26 ± 1 °C, 70 ± 5% relative humidity, and a 14:10 h (light:dark) photoperiod. A total of 400 antennae (200 male antennae and 200 female antennae, pooled together) were dissected from two-day-old virgin adults. These samples were immediately frozen in liquid nitrogen and stored at −80 °C before RNA isolation was conducted.

### 2.2. RNA Extraction, cDNA Library Construction, and Transcriptome Sequencing

Total RNA was extracted using RNAiso Plus reagent (Takara, Dalian, China) following the manufacturer’s instructions. The quality of RNA was assessed using an Agilent 2100 bioanalyzer (Agilent Technologies, Palo Alto, Canada), and the concentration of RNA was determined on a Nanodrop 2000 spectrophotometer (Thermo Scientific, Wilmington, Gemany). For cDNA library construction, mRNA was purified from 20 μg total RNA using oligo (dT) magnetic beads and then sheared into short fragments. The first-strand cDNA was reverse-transcribed from the mRNA fragments with random hexamer primers and MMLV reverse transcriptase (RNaseH-), and the second-strand cDNA was subsequently synthesized using DNA polymerase I and RNaseH. After end-repairing and dA-tailing, the adapters were ligated to the double-stranded cDNA, and the ligation products were subjected to PCR amplification. The amplified products were denatured to single-stranded cDNA by heat treatment, and the final library was generated by cyclization of the single-stranded cDNA using splint oligo and DNA ligase. The cDNA library was sequenced on a BGISEQ-500 platform at Beijing Genomics Institute (BGI-Wuhan, Wuhan, China). The raw data were deposited in the NCBI Sequence Read Archive (SRA) database with accession number SRR8916466.

### 2.3. De Novo Assembly and Functional Annotation

Before de novo assembly, raw reads were subjected to quality control and filtered into clean reads by removing adaptors, low quality reads, and reads containing >10% unknown bases. Transcriptome assembly was performed using the Trinity software (version: v2.0.6) [45]. The longest transcript of each gene was defined as a unigene. To annotate the unigenes, a BLASTX search was performed against the reference sequences in the NCBI non-redundant (NR) and eukaryotic orthologous groups (KOG) databases with a cut-off e-value of 10^−5^. Gene ontology (GO) terms were retrieved by using Blast2GO. The Kyoto Encyclopedia of Genes and Genomes (KEGG) annotation server was used to assign the KEGG pathways of the unigenes [46].

### 2.4. Identification of Genes Encoding ODEs

Candidate genes encoding ODEs were identified by retrieving the transcriptome dataset with the TBLASTN program. The annotated protein sequences of CCEs, GSTs, CYPs, UGTs, and AOXs from other lepidopteran species, including *B. mori*, *P. xylostella*, *M. sexta*, *H. armigera*, *S. littoralis*, *S. exigua*, *S. litura*, *A. lepigone*, *C. pomonella*, *A. transitella*, *C. medinalis,* and *C. suppressalis*, were used as queries. The cut-off e-value was set as 10^−5^. The output was manually checked, and overlapping variants were eliminated. Finally, all the candidates were confirmed by searching against the NCBI NR database using the BLASTX online program (cut-off e-value: 10^−5^).

### 2.5. Bioinformatic Analyses

The open reading frame (ORF) was predicted using ORF Finder (http://www.ncbi.nlm.nih.gov/gorf/gorf.html, accessed on 26 July 2022). The theoretical molecular mass (Mw) and isoelectric point (pI) were obtained using an ExPASy tool (http://web.expasy.org/compute_pi/, accessed on 28 July 2022). Putative signal peptide and transmembrane domain were predicted with SignalP (http://www.cbs.dtu.dk/services/SignalP, accessed on 28 July 2022) and TMHMM (http://www.cbs.dtu.dk/services/TMHMM/, accessed on 28 July 2022), respectively. Functional domains and catalytic residues were predicted by using the NCBI’s Conserved Domain Search (https://www.ncbi.nlm.nih.gov/Structure/cdd/wrpsb.cgi, accessed on 28 July 2022). Deduced protein sequences were aligned using Clustal Omega program (http://www.ebi.ac.uk/tools/msa/clustalo/, accessed on 28 July 2022). The neighbor-joining trees were generated using MEGAX software (version 10.2.0) with 1000 bootstrap replicates [47]. The trees were viewed and edited using the FigTree software v1.4.4 (http://tree.bio.ed.ac.uk/software/figtree/, accessed on 28 July 2022). The GenBank accession numbers of sequences used in the phylogenetic analyses are listed in Table S1.

### 2.6. Expression Profile Analysis

Expression profiles of selected genes in different adult tissues were analyzed using reverse transcription-quantitative PCR (RT-qPCR). Total RNA was isolated from 100 male antennae, 100 female antennae, 60 heads (without antennae; 30 from males, 30 from females, pooled together), 60 abdomens (30 from males and 30 from females, pooled together), and 200 legs (100 from males and 100 from females, pooled together), and reverse transcribed to first-strand cDNA using ReverTra Ace qPCR RT Master Mix with gDNA Remover (Toyobo, Osaka, Japan). Each cDNA sample was diluted to 10 ng/μL using nuclease-free water.

RT-qPCR was performed in a 20 μL reaction mixture containing 10 μL SYBR Green Real-time PCR Master Mix (Toyobo, Osaka, Japan), 1 μL (10 ng) cDNA template, 0.4 μL (0.2 μM) of forward primer, 0.4 μL (0.2 μM) of reverse primer, and 8.2 μL nuclease-free water. Primers are listed in Table S2, and the *ribosomal protein S3* (*RPS3*) and *actin* were used as reference genes. RT-qPCR was carried out in triplicate in 96-well plates and run on a CFX96 Real-time System (Bio-Rad, Hercules, Canada). The thermal cycle parameters were 1 cycle of 95 °C for 2 min, 40 cycles of 95 °C for 5 s, and 60 °C for 20 s. At the end of each thermal cycle, the PCR products were analyzed using a heat-dissociation protocol. To avoid contamination from genomic DNA and reagents, each 96-well plate includes negative controls (no-template control and no reverse transcriptase control) [48]. In addition, the amplified products were confirmed by visualization on an agarose gel, and the products were sequenced to verify the correct amplification of target genes. The RT-qPCR experiment was performed in three biological replications (i.e., the tissue dissection, RNA extraction, and cDNA synthesis were all repeated independently three times) [49]. The relative transcription level of each target gene was first normalized to the levels of the reference genes, then with the mRNA level in the leg. The SATqPCR program was used for calculating the expression levels [50].

### 2.7. Data Statistics

Data analysis was performed using the Data Processing System (DPS) v9.5 software [51]. The differences among various samples were compared by one-way analysis of variance (ANOVA) followed by Tukey’s test. The level of significance was set at *p* < 0.05.

## 3. Results

### 3.1. Unigene Assembly and Functional Annotation

A total of 63.35 Mb raw reads were produced from the *O. furnacalis* antennal transcriptome. After data filtration, 57.28 Mb clean reads were obtained and assembled into 35,056 unigenes with a N50 length of 1995 bp. The unigenes size distribution is shown in Figure S1A. The lengths of 21,015 unigenes (59.95% of all unigenes) were more than 500 bp.

We annotated the *O. furnacalis* unigenes by searching against the NCBI NR database. A total of 21,012 (59.94%) unigenes showed homology to the genes in other insect species. Of these, *H. armigera* was the best match, followed by *A. transitella*, *B. mori*, *Papilio machaon*, and *P. xuthus* (Figure S1B). We performed a GO analysis to better classify the functions of the *O. furnacalis* unigenes. The results showed that 9403 (26.82%) unigenes could be assigned to 3 major categories: biological process (2461 unigenes), cellular component (2807 unigenes), and molecular function (4135 unigenes) (Figure S1C). In each of the three main categories, the terms “cellular process” (1152 unigenes), “cell” (1064 unigenes) and “binding” (1893 unigenes) were dominant, respectively (Figure S1C). We also performed KOG and KEGG annotations. Totals of 14,136 (40.32%) and 16,585 (47.31%) unigenes could be annotated in the KOG and KEGG databases, respectively, based on sequence homology (Figures S2 and S3).

### 3.2. Identification of CCEs

Before this study, Yang et al. (2015) identified 15 *CCE*s (*OfurCCE1* to *OfurCCE15*) from the *O. furnacalis* antennae [39]. Here we retrieved the transcriptome and identified four novel genes (named as *OfurCCE16* to *OfurCCE19*; Table S3). Therefore, the number of *CCE*s is at least 19 in the antennae of *O. furnacalis*. The GenBank accession numbers and detailed information of the four novel *CCE*s are presented in Table S3. The four genes all had complete ORFs and shared ≥ 60% amino acid identities with orthologs from other lepidopteran species. N-terminal signal peptides were predicted for the deduced OfurCCE16 and OfurCCE18 proteins, suggesting that the two proteins could be secreted from the cells (Table S3).

**Figure 1:**
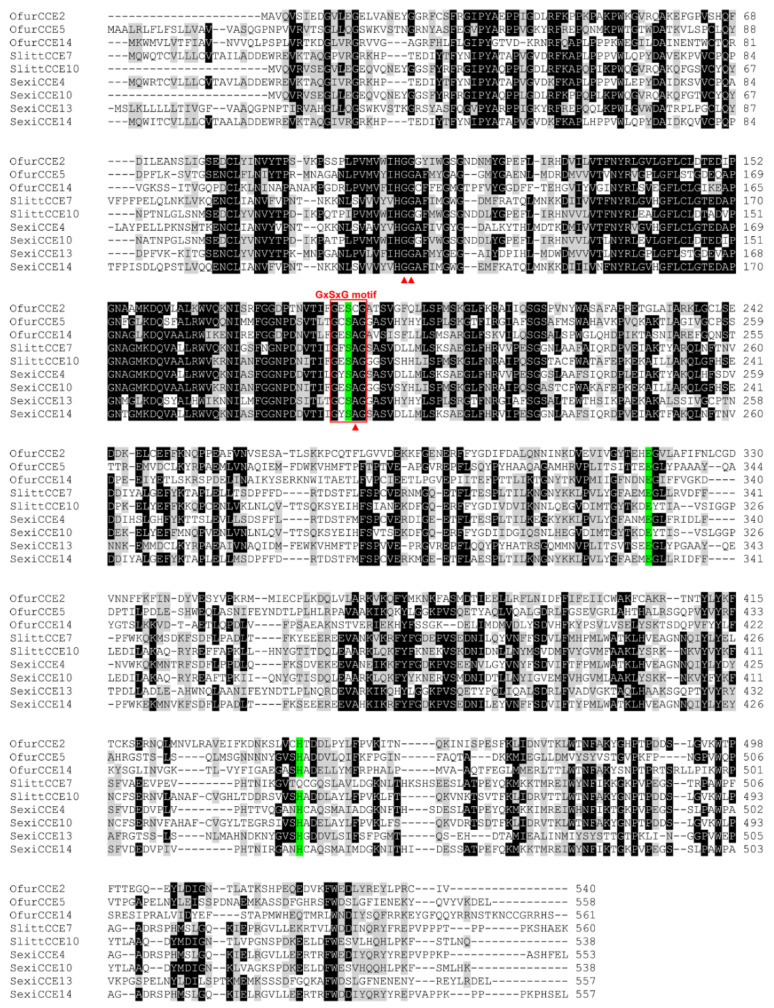
Multiple sequence alignment of the representative CCEs from *Ostrinia furnacalis* (Ofur-prefix), *Spodoptera littoralis* (Slitt), and *S. exigua* (Sexi). Shading represents >50% identity (black) and >50% similarity (grey). OfurCCE2 shares 54% amino acid identity with SlittCCE10, while OfurCCE5 shares 70% amino acid identity with SexiCCE13. The conserved pentapeptide Gly-X-Ser-X-Gly (GxSxG) motif is boxed. The catalytic triad residues are indicated with red triangles. The oxyanion hole-forming residues are highlighted in green color.

**Figure 2:**
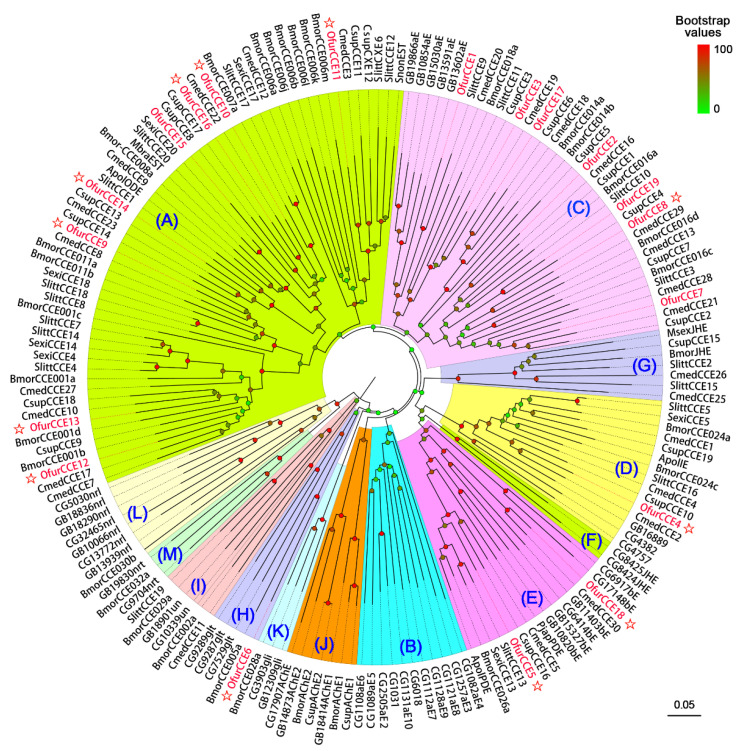
Phylogenetic analysis of CCEs from *Ostrinia furnacalis* and other insect species including *Drosophila melanogaster* (Accession numbers refer to FlyBase (CG-prefix)), *Apis mellifera* (Accession numbers refer to BeeBase (GB-prefix)), *Bombyx mori* (Bmor), *Cnaphalocrocis medinalis* (Cmed), *Spodoptera littoralis* (Slitt), *Spodoptera exigua* (Sexi), *Manduca sexta* (Msex), *Antheraea polyphemus* (Apol), *Mamestra brassicae* (Mbra), *Sesamia nonagrioides* (Snon), *Chilo suppressalis* (Csup), and *Popilia japonica* (Pjap). Bootstrap values are indicated by colors from green (0) to red (100). The CCEs are classified to 13 clades: (**A**) lepidopteran esterases containing odorant degrading esterases; (**B**) dipteran mitochondrial, cytosolic, and secreted esterases; (**C**) lepidopteran and hymenopteran alpha-esterases; (**D**) integument esterases; (**E**) beta esterases and pheromone esterases; (**F**) dipteran JHEs; (**G**) lepidopteran JHEs; (**H**) glutactins; (**I**) unknown function; (**J**) acetylcholinesterases; (**K**) gliotactins; (**L**) neuroligins; (**M**) neurotactins. The *O. furnacalis* CCEs are highlighted in red, and the CCEs with signal peptides are indicated by the star symbols. GenBank accession numbers of sequences used are listed in Table S1.

We analyzed the functional domains and catalytic residues for the 19 antennal CCEs (4 identified in this study and 15 reported by Yang et al. (2015)) by searching the NCBI’s Conserved Domain database. The results showed that most of the CCEs displayed a conserved motif, including the conserved pentapeptide Gly-X-Ser-X-Gly and the oxyanion hole-forming residues Gly, Gly, and Ala (Figure S4). Multiple sequence alignment results also indicated that three representative CCEs (OfurCCE2, OfurCCE5, and OfurCCE14) shared the conserved motifs and residues with their noctuid counterparts known to have an odorant degradation function (Figure 1). A neighbor-joining tree was generated, and the results showed that insect CCEs could be classified into 13 different clades (Figure 2). Eight CCEs (OfurCCE9/10/11 /12/13/14/15/16) were clustered into the “(A) lepidopteran esterases containing odorant degrading esterases” clade, while seven CCEs (OfurCCE1/2/3/7/8/17/19) fell into the “(C) lepidopteran and hymenopteran alpha-esterases” clade (Figure 2). The “(E) beta esterases and pheromone esterases” clade consisted of two OfurCCEs (OfurCCE5 and OfurCCE18), and the “(D) integument esterases” and “(H) glutactins” clades each contained one OfurCCE. In addition, most of the *O. furnacalis* CCEs were clustered with at least one lepidopteran ortholog (Figure 2).

### 3.3. Identification of GSTs

By retrieving the transcriptome dataset, 17 *GST*s (*OfurGSTd1* to *OfurGSTu1*) were identified. All had complete ORFs, and the lengths of the predicted proteins ranged 203 to 286 amino acid residues (Table S3). BLASTX best hit results showed that *OfurGSTd3* shared 98% amino acid identity with a delta-class GST previously identified in *O. furnacalis*; other GSTs shared a 59–93% amino acid identities to other known lepidopteran GSTs (Table S3). Notably, a signal peptide sequence was predicted at the N-terminus of OfurGSTd1 (Table S3). However, this characteristic was not shared with the other GSTs in *O. furnacalis* antennae (Table S3).

**Figure 3:**
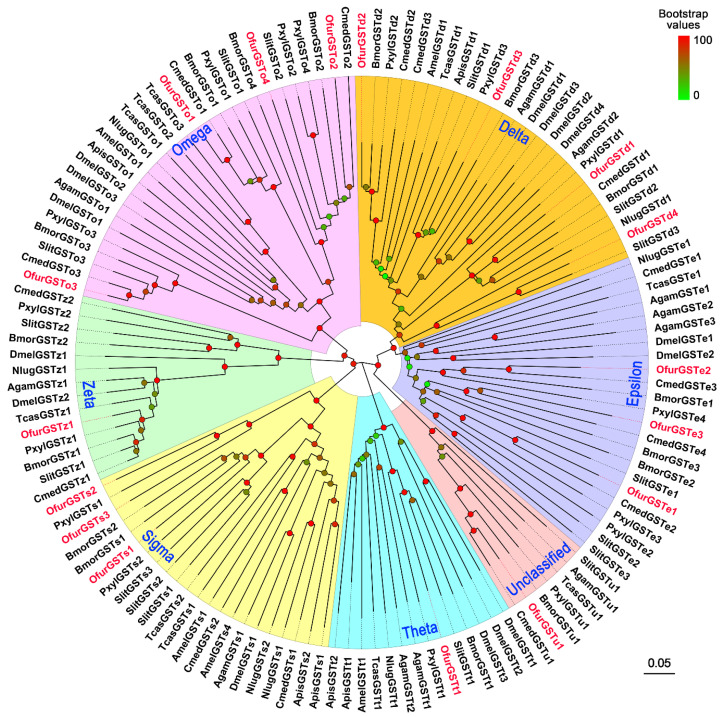
Phylogenetic analysis of GSTs from *Ostrinia furnacalis* (Ofur-prefix), *Bombyx mori* (Bmor), *Plutella xylostella* (Pxyl), *Cnaphalocrocis medinalis* (Cmed), *Spodoptera litura* (Slit), *Drosophila melanogaster* (Dmel), *Anopheles gambiae* (Agam), *Tribolium castaneum* (Tcas), *Apis mellifera* (Amel), *Nilaparvata lugens* (Nlug), and *Acyrthosiphon pisum* (Apis). Bootstrap support values are indicated by coloring from green (0) to red (100) on each node. Insect GSTs are classified into six classes (delta, epsilon, omega, sigma, theta, and zeta) and an “unclassified” clade. The *O. furnacalis* GSTs are colored red. GenBank accession numbers of sequences used are listed in Table S1.

Analysis of the conserved domain revealed glutathione binding sites (G-sites) in the N-terminal regions of nine of the OfurGST proteins but not in OfurGSTe1, OfurGSTe3, OfurGSTo1, OfurGSTo2, OfurGSTo3, OfurGSTo4, OfurGSTz1, or OfurGSTu1, and substrate binding sites (H-sites) in the C-terminal region of 14 OfurGSTs but not in OfurGSTe3, OfurGSTo2, or OfurGSTz1 (Figure S5). Phylogenetic analysis showed that the 17 OfurGSTs were segregated into 7 clades, namely, delta (4 OfurGSTs), epsilon (3), omega (4), sigma (3), theta (1), and zeta (1), and an “unclassified” clade (one) (Figure 3). In this tree, OfurGSTs were found to be more closely related to lepidopteran GSTs than to GSTs from non-lepidopteran species (Figure 3).

### 3.4. Identification of CYPs

We identified 24 *CYP*s from the transcriptome. All of the *CYP*s had complete ORFs, and the lengths of the deduced CYP proteins ranged from 457 to 560 amino acids (Table S3). The transmembrane domains were predicted in the N-termini of 21 CYP proteins but not in OfurCYP305B1, OfurCYP306A1, or OfurCYP333A20 (Table S3). BLASTX best hit results showed that all the *O. furnacalis* CYPs shared quite high (57–92%) amino acid identities with CYPs from other lepidopteran species, including *C. suppressalis*, *C. medinalis*, and *H. armigera* (Table S3).

**Figure 4:**
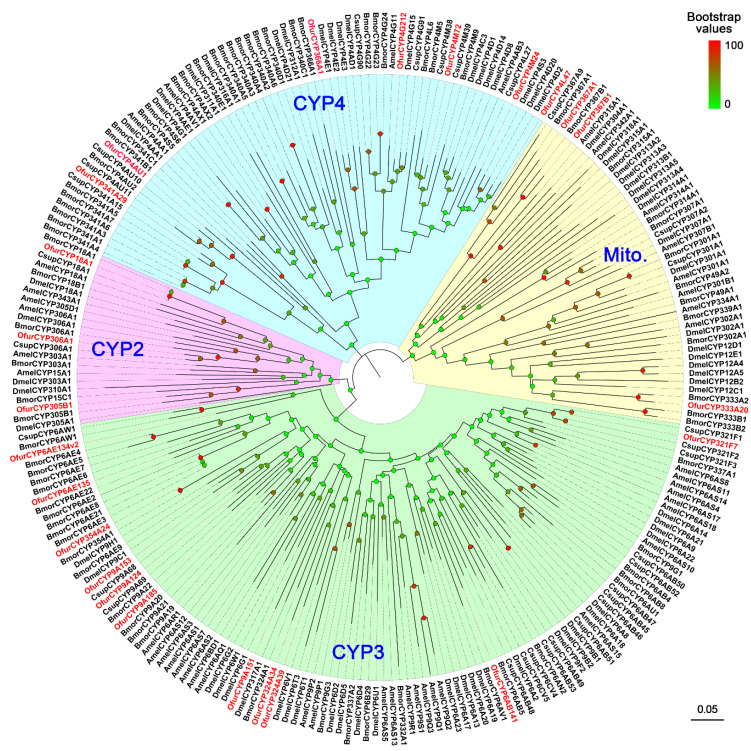
Phylogenetic analysis of CYPs from *Ostrinia furnacalis* (Ofur-prefix), *Drosophila melanogaster* (Dmel), *Apis mellifera* (Amel), *Bombyx mori* (Bmor), and *Chilo suppressalis* (Csup). Bootstrap values are indicated by coloring from green (0) to red (100) on each node. Insect CYPs are classified into four major clans (CYP2, CYP3, CYP4, and mitochondrial). The *O. furnacalis* CYPs are colored red. CYP sequences used in this analysis are listed in Table S1.

Multiple sequence alignment analysis revealed that these CYPs had five conserved domains, including helix-C, helix-I, helix-K, PERF, and heme-binding motifs (Figure S6). In the phylogenetic analysis, we found that insect CYPs were divided into four major clans: CYP2, CYP3, CYP4, and mitochondrial clans, and were subdivided into different families (Figure 4). In this tree, most of the *O. furnacalis* CYPs fell into the CYP3 (11 genes) and CYP4 (7 genes) clans. In particular, the CYP4 and CYP6 families—two well-known detoxification families—included five and three OfurCYPs, respectively (Figure 4).

### 3.5. Identification of UGTs and AOXs

**Figure 5:**
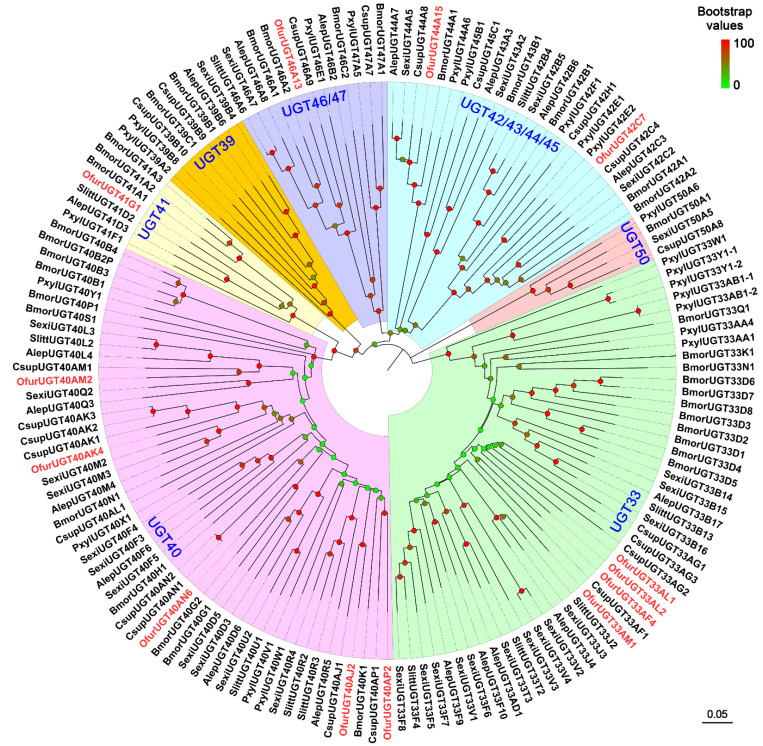
Phylogenetic analysis of UGTs from *Ostrinia furnacalis* (Ofur-prefix), *Bombyx mori* (Bmor), *Spodoptera littoralis* (Slitt), *Spodoptera exigua* (Sexi), *Plutella xylostella* (Pxyl), *Athetis lepigone* (Alep), and *Chilo suppressalis* (Csup). Bootstrap values are indicated by coloring from green (0) to red (100) on each node. The *O. furnacalis* UGTs are colored red. UGT sequences used in this analysis are listed in Table S1.

We identified 13 putative *UGT*s from the antennae of *O. furnacalis*. Of these, 11 sequences had complete ORFs, and the lengths of the deduced proteins ranged from 514 to 531 amino acid residues; the other 2 (*OfurUGT33AF4* and *OfurUGT40AP2*) were partial sequences encoding 454 and 509 amino acids, respectively (Table S3). Of the 13 OfurUGT proteins, 10 possessed a signal peptide at the N-terminus, and 12 possessed a transmembrane domain at the C-terminus (Table S3). All the OfurUGTs shared ≥ 56% amino acid identity with their respective orthologs from other lepidopterans (Table S3). Sequence analysis revealed two sugar donor-binding regions (DBR1 and DBR2) in all the OfurUGT proteins; several key residues that are involved in the interaction of sugar donors were also found in the DBR1 and DBR2 regions (Figure S7). Phylogenetic analysis showed that the 13 *O. furnacalis* UGTs were clustered into six families (Figure 5). In this tree, there were five OfurUGTs in the UGT40 family and four OfurUGTs in the UGT33 family. There was only one OfurUGT each in the UGT41, UGT42, UGT44, and UGT46 families (Figure 5).

**Figure 6:**
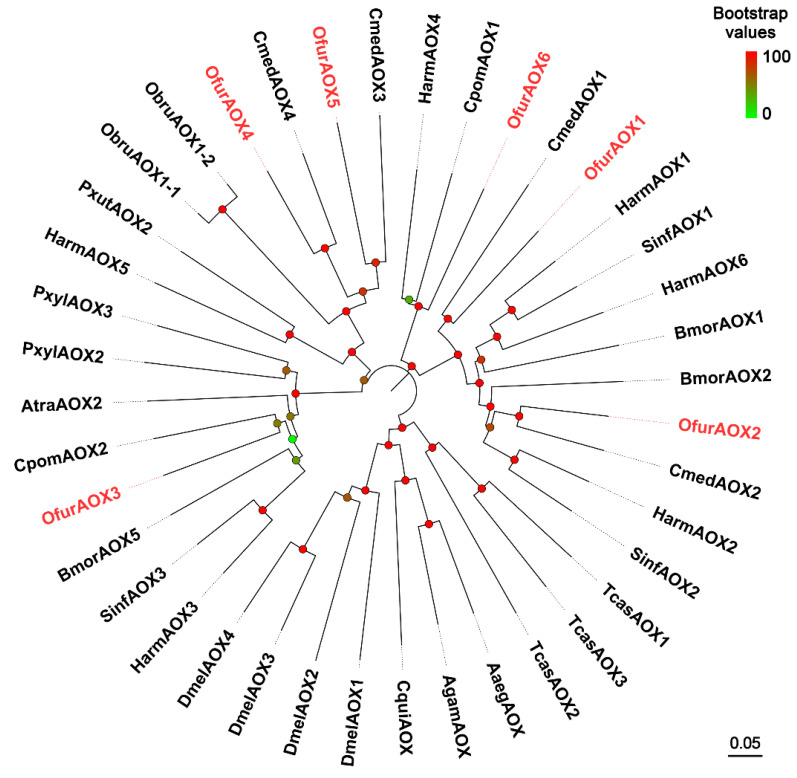
Phylogenetic analysis of AOXs from Ostrinia furnacalis (Ofur-prefix), Helicoverpa armigera (Harm), Cnaphalocrocis medinalis (Cmed), Amyelois transitella (Atra), Bombyx mori (Bmor), Sesamia inferens (Sinf), Cydia pomonella (Cpom), Plutella xylostella (Pxyl), Papilio xuthus (Pxut), Operophtera brumata (Obru), Culex quinquefasciatus (Cqui), Aedes aegypti (Aaeg), Anopheles gambiae (Agam), Drosophila melanogaster (Dmel), and Tribolium castaneum (Tcas). Bootstrap values are indicated by coloring from green (0) to red (100). The O. furnacalis AOXs are colored red. AOX sequences used in this analysis are listed in Table S1.

We identified six putative *AOX*s in the antennal transcriptome (Table S3). The deduced OfurAOX proteins shared 99–100% amino acid identity with AOXs previously identified in *O. furnacalis* antennae [39]. Phylogenetic analysis showed that OfurAOX1, OfurAOX2, OfurAOX4, and OfurAOX5 had close relationships with their respective orthologs from *C. medinalis*, whereas OfurAOX3 was located on a branch with *C. pomonella* AOX2, and OfurAOX6 fell into a branch with *C. pomonella* AOX1 and *H. armigera* AOX4 (Figure 6).

### 3.6. Expression Profiles of O. furnacalis CCEs

**Figure 7:**
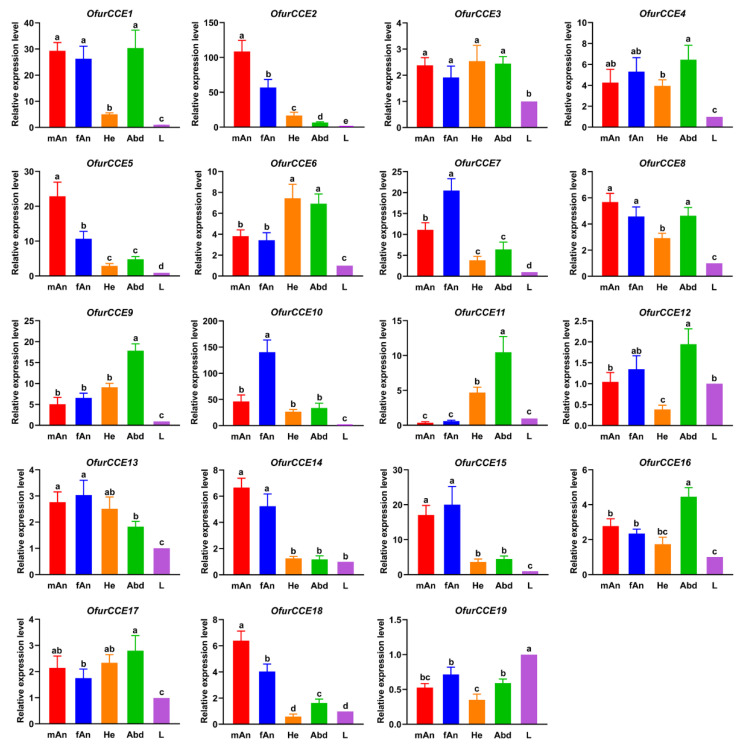
Relative expression levels of *Ostrinia furnacalis CCE*s in various adult tissues. mAn, male antennae; fAn, female antennae; He, heads; Abd, abdomens; L, legs. Results are means of three biological replicates ± standard error (SE). The average expression value of each *CCE* gene in the leg was set to one. Different lowercase letters indicate significant variation in transcription among different tissues (one-way ANOVA with Tukey’s test, *p* < 0.05).

Tissue- and sex-biased expression profiles of 19 *OfurCCE*s were investigated by RT-qPCR (Figure 7). The results showed that none of the *OfurCCE* genes were antennae-specific, and that several of the *OfurCCE*s were poorly expressed in all tissues tested (e.g., *OfurCCE19*, where expression was <1 in all tissues). We observed that seven genes (*OfurCCE2*/*5*/*7*/*10*/*14*/*15*/*18*) were highly expressed in the antennae. Of these, *OfurCCE2*, *OfurCCE5*, and *OfurCCE18* were enriched in male antennae, while *OfurCCE7* and *OfurCCE10* were enriched in female antennae. The remaining *OfurCCE14* and *OfurCCE15* were expressed at near-equal amounts in antennae of both females and males (Figure 7). We also observed that several *OfurCCE*s were highly expressed in non-olfactory tissues. For example, *OfurCCE9*, *OfurCCE11*, and *OfurCCE16* had the highest expression levels in the abdomen (Figure 7). In addition, several *OfurCCE*s were expressed in both antennae and non-olfactory tissues, e.g., *OfurCCE1* in male and female antennae as well as in abdomen; *OfurCCE3* in male antennae, female antennae, head, and abdomen (Figure 7).

## 4. Discussion

The goal of this study was to identify putative ODEs in the antennae of *O. furnacalis* that might be involved in the degradation of odorant molecules and thereby in the regulation of the behavior of this insect species. To achieve this goal, we performed a transcriptomic analysis. Previously, two antennal transcriptomes were constructed for *O. furnacalis* [38,39]. However, only a small number of ODEs were identified [39]. In the present study, 79 genes encoding the ODEs were discovered from the antennal transcriptome of *O. furnacalis*. Our findings will provide a resource for functional study of these ODEs and for screening candidate genes for novel pest control strategies. For example, two CCE genes were found to be highly expressed in the male antennae of *G. molesta*, and knockdown of either gene by RNAi not only affects the electroantennogram (EAG) responses of male moths to sex pheromones, but also inhibits the searching behavior of males for females [52].

Before this study, Yang et al. (2015) identified 15 *CCE*s from the *O. furnacalis* antennae [39]. In this study, we identified four novel *OfurCCE*s. Therefore, the total number of *CCE*s in the *O. furnacalis* antennae is at least 19. This number is less than that in *Ectropis obliqua* (35 genes) [53] and *D. melanogaster* (25 in male antennae and 26 in female antennae) [9], but is comparable to those in *A. lepigone* (20 genes) [42], *S. littoralis* (19 genes) [43], *C. suppressalis* (19 genes) [41], *C. medinalis* (18 genes) [35], and *C. pomonella* (12 genes) [40]. Conserved sequence motifs were found within the 19 OfurCCE proteins, including the pentapeptide Gly-X-Ser-X-Gly and oxyanion hole residues (Gly, Gly, and Ala) that are critical for enzymatic activity [5]. Phylogenetic analysis showed that eight OfurCCEs were clustered into the “Lepidopteran esterases containing odorant degrading esterases” clade. This clade consisted of several CCEs associated with odorant degradation function, including the ApolODE from *A. polyphemus* [54], MbraEST from *Mamestra brassicae* [55], SlittCCE7 from *S. littoralis* [12], and SexiCCE4 and SexiCCE14 from *S. exigua* [14,15]. Seven OfurCCEs fell into the “Lepidopteran and hymenopteran alpha-esterases” clade. CCEs in this clade may be involved in odorant inactivation. The *S. littoralis* CCE10 (SlittCCE10), an antennae-specific esterase hydrolyzing a green leaf volatile (Z3-6:OAc) and two sex pheromone components (Z9E11-14:OAc and Z9E12-14:OAc) [11], was also in this clade. Notably, two OfurCCEs (OfurCCE5 and OfurCCE18) were clustered into the “Beta esterases and pheromone esterases” clade together with two well-characterized pheromone-degrading enzymes, ApolPDE of *A. polyphemus* and PjapPDE of *P. japonica* [7,10]. This clade also included the *S. exigua* CCE13 (SexiCCE13). The recombinant SexiCCE13 enzyme showed high catalytic activity to a variety of acetate substrates, including the sex pheromones, their analogs, and some common plant odorants [13]. The phylogenetic diversity of these *O. furnacalis* CCEs suggested that they may play important roles in the metabolism of odorants.

We identified a total of 17 *OfurGST*s from the *O. furnacalis* antennae. Although the number was less than that in *S. littoralis* antennae (33 genes) [56], it was comparable to those in other lepidopteran species such as *Streltzoviella insularis* (17 genes) [57], *C. suppressalis* (16 genes) [41], and *C. pomonella* (14 genes) [58]. Most of the deduced OfurGST proteins contained a G-site and an H-site, suggesting that these genes encoded functional enzymes. Phylogenetic analysis showed that OfurGSTd1, OfurGSTd2, OfurGSTd3, and OfurGSTd4 were clustered into the delta clade. Delta is the most common class in insects, and several members from this class are involved in olfaction by degrading odorants. For example, two well-characterized olfactory-related GSTs in *M. sexta* (GST-msolf1) and *G. molesta* (GmolGSTd1) belong to the delta class [19,21]. Additionally, a number of delta class GSTs were found to be specifically or highly expressed in the antennae of *A. transitella*, *B. mori*, and *S. littoralis*, suggesting their involvement in odorant inactivation [20,56,59]. Notably, we found a signal peptide at the N-terminus of OfurGSTd1, indicating that the protein may be secreted. Durand et al. (2018) analyzed the signal peptide signatures in GSTs from different insect orders and found that this structure is a common feature and is not restricted to delta GSTs [56]. Thus, these GSTs could be secreted into the sensillar lymph and interact directly with odorants and/or toxic molecules. In addition to the delta class, OfurGSTs were grouped into other classes, including epsilon, omega, sigma, theta, and zeta, and an “unclassified” clade. GSTs in these classes also play important roles in insecticide detoxification and oxidative stress protection [18], and they are expected to have an odorant degradation function.

We identified 24 *OfurCYP*s from the antennal transcriptome. The total number of antennal *CYP*s varies greatly among different insect species; there are 16, 37, 49, 58, and 92 CYPs expressed in the antennae of *S. nonagrioides* [60], *S. littoralis* [44], *Epiphyas postvittana* [61], *D. melanogaster* [9], and *Locusta migratoria* [62], respectively. The number of *OfurCYP*s identified in this study was in a comparable range to the number in other insect species. Phylogenetic analysis has shown that these OfurCYPs are widely distributed in various clans and families. Of these, the CYP4 and CYP6 families are well known for their function in detoxification of toxic compounds [22]. Recently, several members from the two families were found to be involved in the olfaction process. In *D. melanogaster*, the *CYP6A20* gene is expressed in the support cells in the pheromone-sensing olfactory sensilla and regulates pheromone sensitivity [63]. Furthermore, the antennae-enriched CYP4G11 is able to metabolize short-chain aldehydes in *A. mellifera* [25]. Moreover, knockdown of the *CYP4L4* in *S. litura* antennae significantly reduced the olfactory response to the sex pheromone [64]. We found that five and three OfurCYPs were grouped into CYP4 and CYP6 families, respectively. These genes may have potential functions in metabolism of odorants. Apart from CYP4 and CYP6 families, members belonging to other families are also involved in odorant clearance. In *D. ponderosae*, the antennae-specific CYP345E2 can rapidly degrade monoterpene volatiles from the host [27]. However, the ortholog of *D. ponderosae* CYP345E2 was not identified in the *O. furnacalis* antennae. In *B. mori*, insecticide exposure resulted in a coordinated expression of CYPs, chemosensory proteins (CSPs), and odorant-binding proteins (OBPs) in a tissue-dependent manner, suggesting an involvement of these two binding protein families (CSPs and OBPs) in the degradative process by interacting with CYPs [65,66]. Therefore, in future studies, it will be perhaps important to investigate the correlations of CYP and CSP/OBP interaction not only with odorants but also with xenobiotics. The tissue distribution of ODEs (this paper) is not restricted to the antennae, as found for CSPs and OBPs [65,66].

We identified only small numbers of *OfurUGT*s (13 genes) and *OfurAOX*s (6 genes) from the antennae transcriptome. Our numbers are comparable to those from other insect species such as *D. melanogaster* [9], *S. littoralis* [67], *A. lepigone* [31], *B. mori* [68], *C. medinalis* [35], and *H. armigera* [34]. Previous research revealed that the insect UGTs and AOXs play important roles in the inactivation of odorant signals. For example, the ability to discriminate sex pheromone (cVA) is regulated by the expression of *UGT36E1* in the antennae of *D. melanogaster* [29]. Moreover, a diversity of aldehydes (including sex pheromones and plant volatiles) can be degraded directly by antennal AOXs in various lepidopteran insects [32,33,69]. It is possible that the *OfurUGT*s and *OfurAOX*s are involved in the degradation of semiochemicals. However, functional studies are needed to test this hypothesis.

The *O. furnacalis* adults can perceive a wide range of ester odorants, including the sex pheromone components Z12-14:OAc and E12-14:OAc, as well as various volatile esters from host plants [36,37]. These ester compounds may be degraded by CCEs expressed in the antennae. Thus, elucidation of the transcriptional patterns of *OfurCCE*s in different adult tissues may help to predict their olfactory roles. The results indicated that the transcription of seven *OfurCCE* genes was antennae-enriched. Of these, *OfurCCE2*, *OfurCCE5* and *OfurCCE18* were primarily expressed in the male antennae, while *OfurCCE7* and *OfurCCE10* were mainly expressed in the female antennae. These genes may encode active enzymes responsible for the degradation of ester odorants. A number of antennal CCEs have been functionally characterized [7,8,9,10,11,12,13,14,15,16,17,52]. The ApolPDE from *A. polyphemus* and PjapPDE from *P. japonica* are both restrictively expressed in the male antennae and are capable of degrading intraspecific sex pheromones [7,10]. Furthermore, metabolism of sex pheromones and plant volatiles by antennal CCEs (SlCXE7, SlCXE10, SexiCXE4, and SexiCXE14) was found in *S. littoralis* and *S. exigua* [11,12,14,15]. However, male- or female-biased expression of CCEs does not necessarily confer specific activity to sex pheromones or to plant volatiles. For example, SexiCCE10, which is highly expressed in both male and female antennae, displays high specificity for plant volatiles but no activity for sex pheromone constituents [16]. Therefore, heterogeneous expression and functional assays are needed to test the odorant degradation functions of the OfurCCEs identified.

In addition to the antennae-enriched *OfurCCE*s, several *OfurCCE*s were found to be highly expressed in non-olfactory tissues or were expressed in both antennae and non-olfactory tissues. These CCEs may have hydrolytic activity with harmful xenobiotic compounds in non-olfactory tissues, or they may have a dual function that not only includes inactivation of semiochemicals in antennae but also degradation of xenobiotics in other tissues [4,17]. Similar expression patterns have also been reported in other insect species [40,41,42,43].

In this study, we analyzed the transcriptional profiles of *OfurCCE* genes in different adult tissues. By using RT-qPCR, we were able to show that none of these *OfurCCE*s are expressed specifically in the antennae. We did not test *OfurCCE* expression in the epidermis or in metabolic organs such as the midgut and fat body. CYPs, GSTs, UGTs, and AOXs, in addition to CCEs, may also play important roles in odorant degradation [4]. Therefore, further studies are needed to provide comprehensive expression data of *OfurCCE* and other *ODE* genes across tissues and throughout insect development.

## 5. Conclusions

In conclusion, using a transcriptome sequencing approach, we identified a total of 79 candidate genes encoding ODEs (19 CCEs, 17 GSTs, 24 CYPs, 13 UGTs, and 6 AOXs) from an important lepidopteran pest, *O. furnacalis*. The CCE genes were differentially expressed in antennae and non-olfactory tissues, indicating a potential involvement in olfactory signal inactivation and other physiological processes in this insect species. The present study paves the way for further research aimed not only at understanding the function of ODEs in *O. furnacalis,* but also at developing novel management strategies using ODEs as potential targets for disrupting insect olfactory behaviors.

## Data Availability

The data presented in this study are available in Supplementary Materials.

## Supplementary Materials

The following supporting information can be downloaded at: https://www.mdpi.com/article/10.3390/insects13111027/s1, Figure S1. Summary of the *Ostrinia furnacalis* unigene assembly, Figure S2. KOG classification of the *O. furnacalis* unigenes, Figure S3. KEGG classification of the *O. furnacalis* unigenes, Figure S4. Catalytic motifs of the *O. furnacalis* CCEs, Figure S5. Predicted glutathione binding sites (G-sites) and substrate binding sites (H-sites) of the *O. furnacalis* GSTs, Figure S6. Functional domains of the *O. furnacalis* CYPs, Figure S7. Alignment of the functional domains of *O. furnacalis* UGTs, Table S1. GenBank accession numbers of genes used in phylogenetic analyses, Table S2. Primers used in RT-qPCR, Table S3. Putative odorant-degrading enzymes identified in *O. furnacalis* antennae.
