# Supplementary material for: Transcriptomic Identification and Expression Profile Analysis of Odorant-Degrading Enzymes from the Asian Corn Borer Moth, Ostrinia furnacalis
Source: Insects. 2022 Nov 6;13(11):1027. doi: 10.3390/insects13111027 (PMC9697913; doi:10.3390/insects13111027)
Supplement: Supplementary file 1 [file insects-13-01027-s001.zip › Suppl Figures S1-S7.pdf]

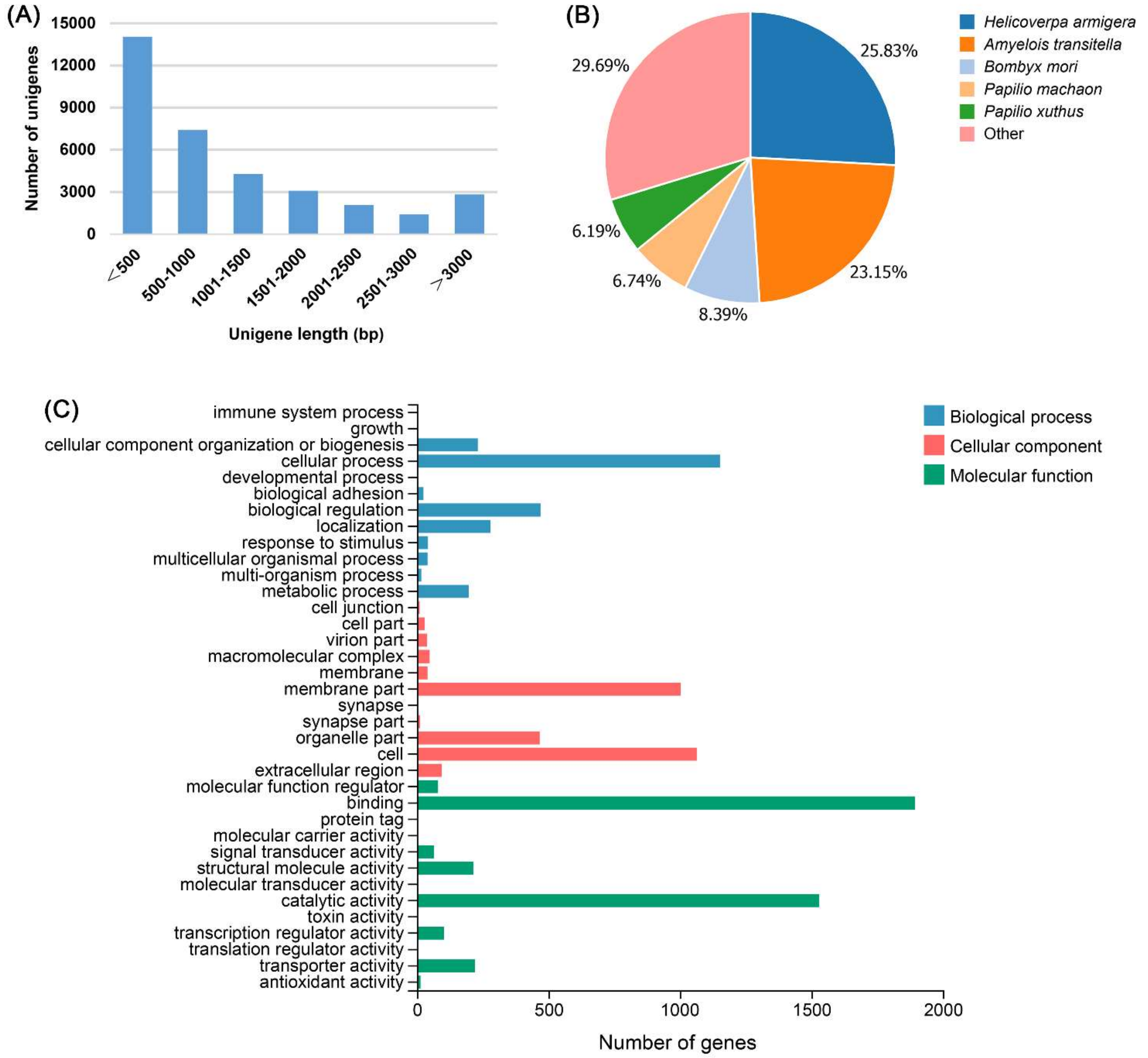

Figure S1. Summary of the *Ostrinia furnacalis* unigene assembly. (A) Unigene size distribution. All of the unigene sizes were calculated. (B) Species distribution of the unigenes based on BLASTX results. (C) GO classification of the unigenes.

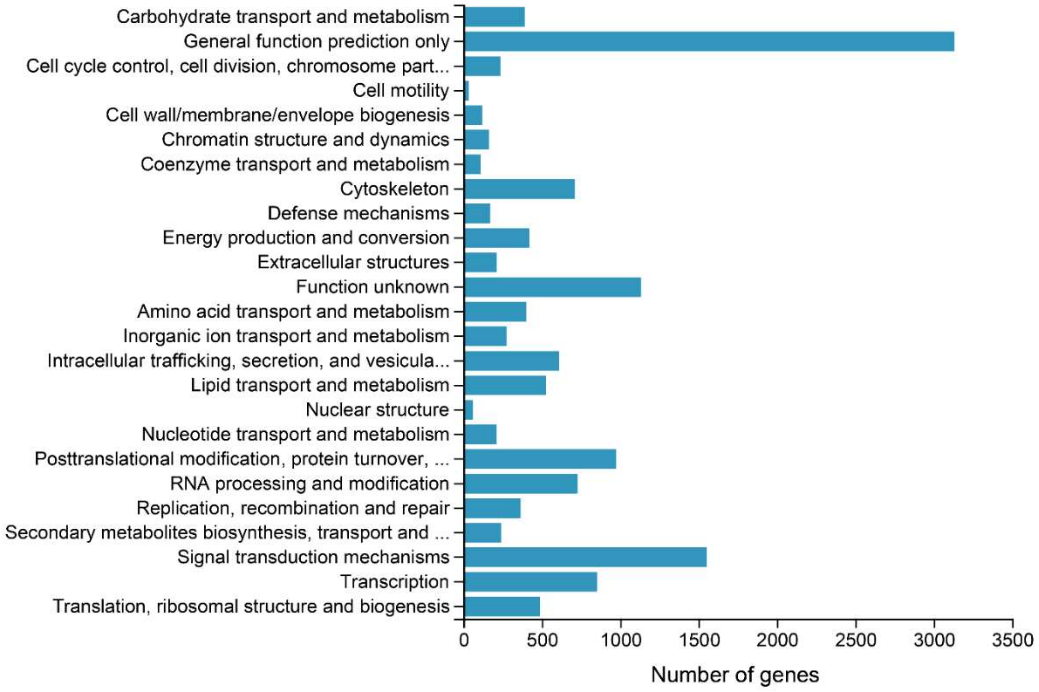

Figure S2. KOG classification of the *O. furnacalis* unigenes.

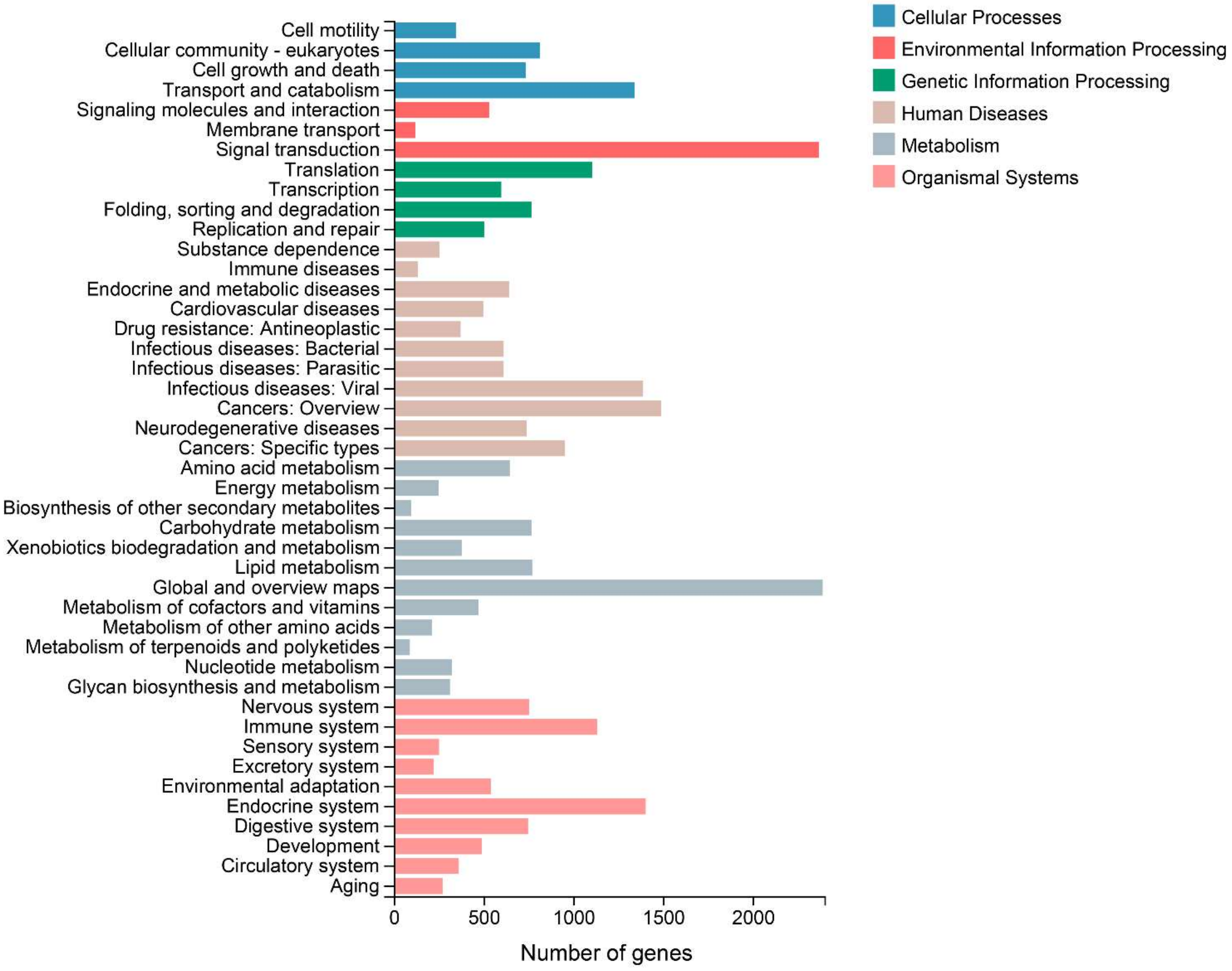

Figure S3. KEGG classification of the *O. furnacalis* unigenes.

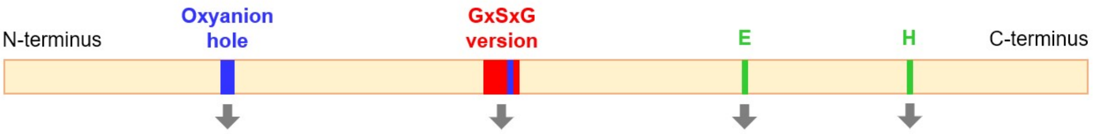

OfurCCE1	GTS	GSSSG	+	+
OfurCCE2	GGC	GESCG	+	+
OfurCCE3	GGA	GVSAG	+	+
OfurCCE4	SGA	GCSAG	+	+
OfurCCE5	GGA	GCSAG	+	+
OfurCCE6	GGF	GQGFG	+	+
OfurCCE7	GGA	GESAG	+	+
OfurCCE8	GGA	GESAG	+	+
OfurCCE9	GGA	GESAG	+	+
OfurCCE10	GGA	GESAG	+	+
OfurCCE11	GGY	GESYG	+	+
OfurCCE12	GGA	GYSAG	+	+
OfurCCE13	GGA	GYSAG	+	+
OfurCCE14	GGA	GESAG	+	+
OfurCCE15	GGA	GMSAG	+	+
OfurCCE16	GGA	GESAG	+	+
OfurCCE17	GGA	GESAG	+	+
OfurCCE18	GGA	GESAG	+	+
OfurCCE19	GGA	GCSAG	+	+

Figure S4. Catalytic motifs of the *O. furnacalis* CCEs.

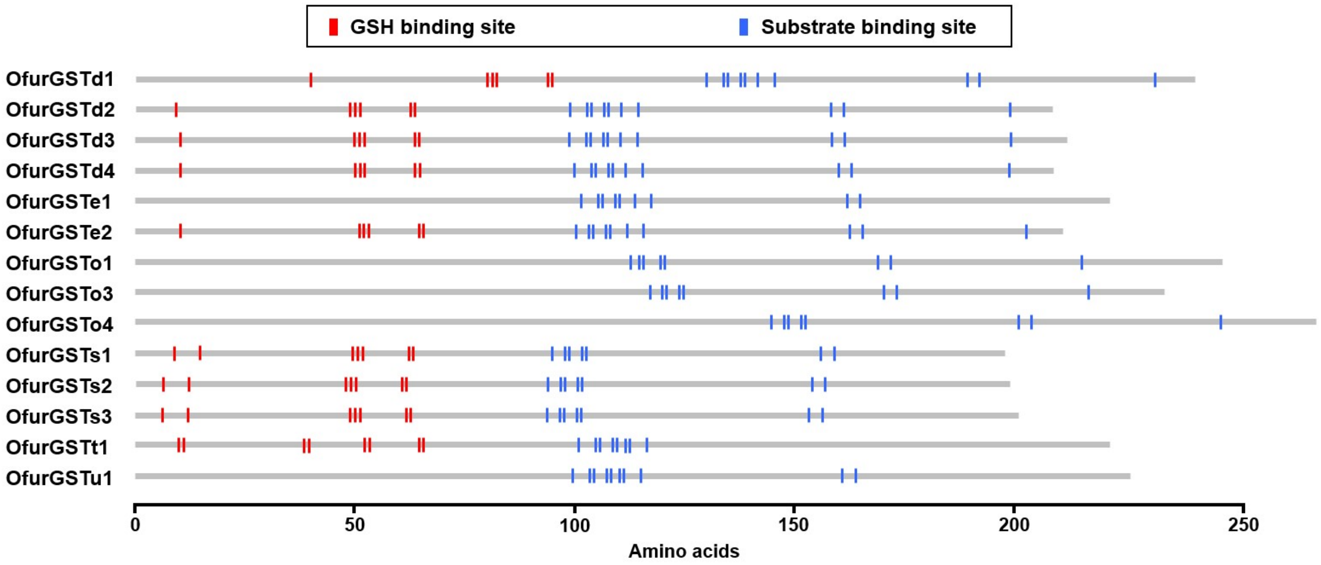

Figure S5. Predicted glutathione binding sites (G-sites) and substrate binding sites (H-sites) of the *O. furnacalis* GSTs. OfurGSTe3, OfurGSTo2 and OfurGSTz1 are not shown in this figure since no G-sites and H-sites are predicted in these proteins.

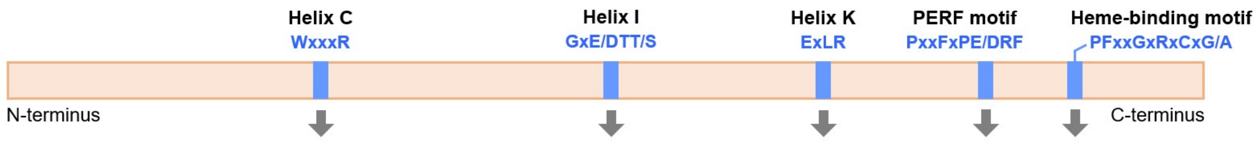

OfurCYP4G212	WRSHR	GHDTT	ETLR	PTKFNPDNF	PFSAGPRSCVG
OfurCYP4L47	WKAHR	GHDTT	ESLR	PLEFRPDRF	AFSAGPRNCIG
OfurCYP4M24	WQQRR	GHDTT	ESLR	ALKFDPDRF	PFSAGPRNCIG
OfurCYP4M72	WQQRR	GHDTT	ESMR	PTQFIPDRF	PFSAGPRNCIG
OfurCYP4AU1	WKVHR	GNDTT	ETMR	ADCFNPDRF	PFSHGTRNCIG
OfurCYP6AB141	WKMLR	GFETS	ESMR	PKEFRPERH	PFGEGPRNCIG
OfurCYP6AE134v2	WKVVR	GYETS	EALR	PEVYRPDRF	PFGDGPRLCIG
OfurCYP6AE135	WKVVR	GYETS	EAMR	PEVYRPQRF	PFGDGPRICIG
OfurCYP9A151	WRNMR	GFETV	ELMR	PDKFDPERF	PFGIGPRNCIG
OfurCYP9A153	WKDMR	GFETV	EVLR	PLRFDPERF	PFGLGPRNCIG
OfurCYP9A124	WKDMR	GFEGI	EVLR	PDKFDPERF	PFGLGPRNCIG
OfurCYP9A185	WKDMR	GFETV	EVLR	PNKFDPERF	PFGLGPRNCIG
OfurCYP18A1	WKSQR	GMETI	ETLR	PKQFNPSRF	PFGVGRRMCLG
OfurCYP305B1	WREHR	GSQTT	EVQR	PHEFKPERF	PFGLGRRRCPG
OfurCYP306A1	WKDQR	GLDTT	ETQR	PEEFRPSRF	PFQTGKRMCPG
OfurCYP321F7	WKLMR	GVEPA	ESLR	PEVFDPERF	PFGYGHRACIG
OfurCYP324A34	WSMIR	GFDTS	ETLR	PMKFDPDRF	PFGEGPRNCIG
OfurCYP324A39	WSSLR	GFDTS	EALR	PDNFDPDRF	PFGQGPRNCIG
OfurCYP333A20	WRDFR	GIDTV	ESMR	AKEFIPERW	PFGFGVRSCIG
OfurCYP341A29	WRRRR	GTDTS	ESLR	ADSFDPDRF	PFSQGPRNCLG
OfurCYP354A24	WKGVR	GYETS	ETLR	PEEIRPERF	AFGVGPRNCIG
OfurCYP366A1	WKHNI	GQETV	ETLR	VLSYRPERW	AFSYGRRSCIG
OfurCYP367A1	WRPHR	SQEAA	ETLR	PLKAKPERF	PFSLGPMDCLG
OfurCYP367B1	WRKHR	SQEAS	EVLR	PDAFDPDRF	PFSLGPMDCLG

Figure S6. Functional domains of the *O. furnacalis* CYPs.

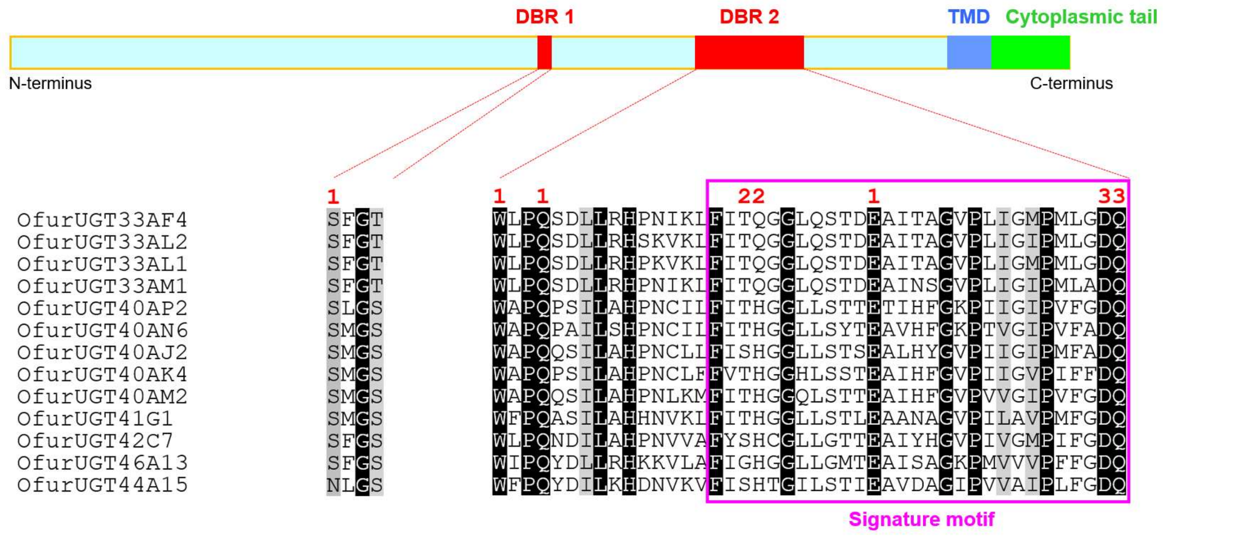

Figure S7. Alignment of the functional domains of *O. furnacalis* UGTs. Upper panel: schematic drawing of *O. furnacalis* UGTs. Two sugar donor-binding regions (DBR1 and DBR2), transmembrane domain (TMD) and cytoplasmic tail are shown. Lower panel: alignment of the DBR1 and DBR2 regions. Key amino acid residues interacting with the sugar donor are numbered 1, 2 and 3 in red (1, nucleotide interacting residues; 2, phosphate interacting residues; 3, glucoside interacting residues).
